# A True Double Optic Disc

**DOI:** 10.1155/crop/6157555

**Published:** 2026-03-08

**Authors:** Mesut Tekerek, Nur Demir

**Affiliations:** ^1^ Ophthalmology Department, Sultan Abdulhamid Han Educational and Research Hospital, Istanbul Provincial Directorate of Health, Istanbul, Turkey

## Abstract

A true double optic disc with two separate vasculatures is an extremely rare condition. The division of the optic nerve into two distinct fascicles is presumed to occur during early stages of embryonic development. Two separate optic discs or one optic disc with two retinal vasculatures are variants of a true double optic disc. This case report highlights a rare form of a true double optic disc with two retinal vasculatures in the same optic disc cup with multimodal imaging.

## 1. Introduction

The presence of a true double optic disc is an extremely rare ophthalmological anomaly [1, 2]. It is characterized by two separate optic discs, each with its own retinal vasculature. There are a few structural variations. Two fully developed optic nerve heads with separate vasculature or an accessory optic disc with separate vasculature could be detected. The rarest morphological variant involves the emergence of a second retinal vasculature from the same optic cup [2]. Colobomas and chorioretinal scars can sometimes mimic a double optic disc; however, in these conditions, the retinal vessels usually traverse the lesion rather than emerging from it [3].

## 2. Case

**Figure 1:**
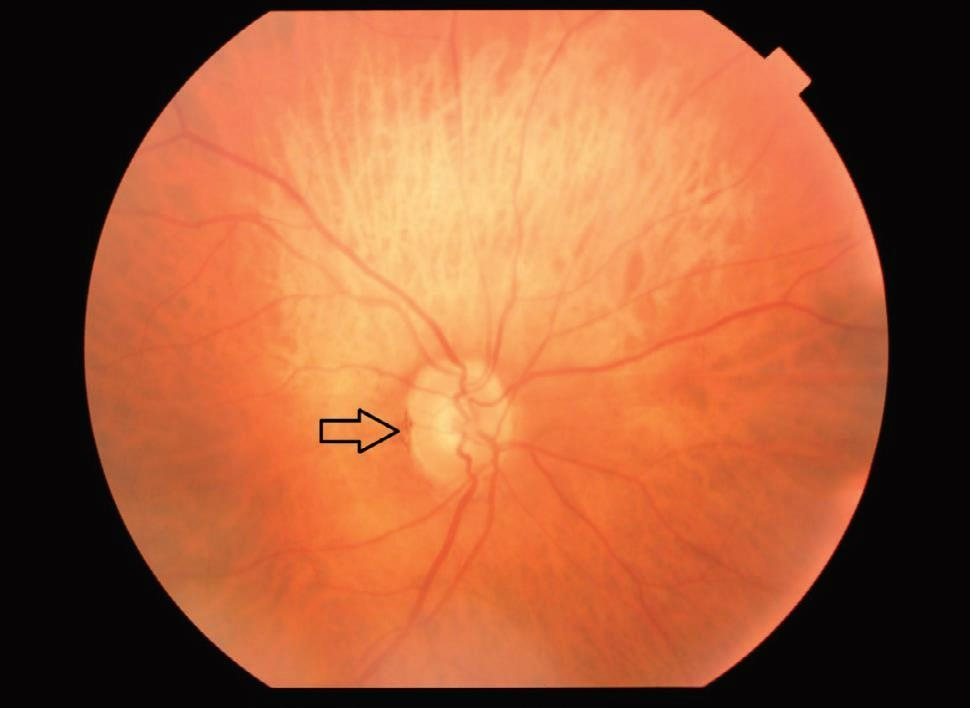
The right eye shows a fused double optic disc appearance, with a notch marked by an arrow.

**Figure 2:**
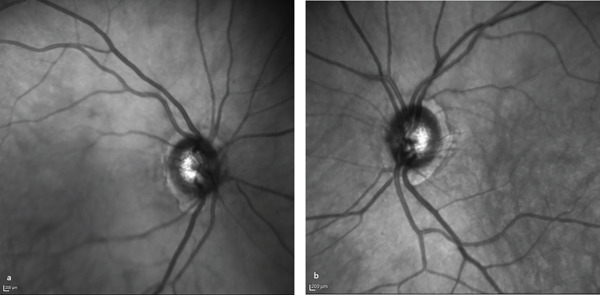
Infrared imaging of the right (a) and left (b) eyes.

**Figure 3:**
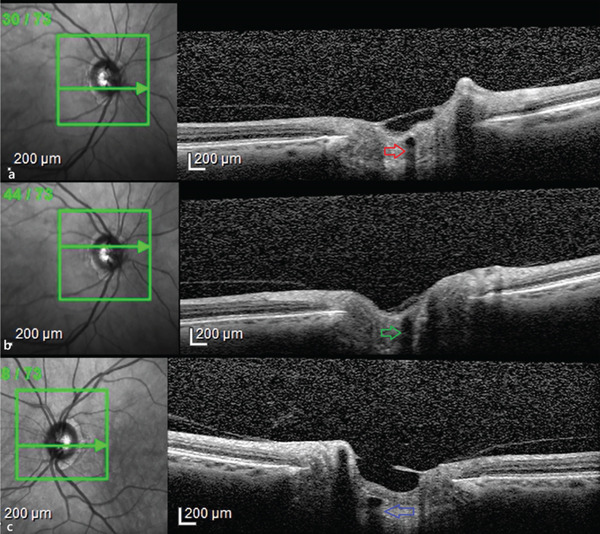
Optical coherence tomography examination of the optic nerve head. (a, b) The right eye optic nerve head shows two central vessel reflections marked with arrows. (c) A single central vessel reflection on the left eye.

**Figure 4:**
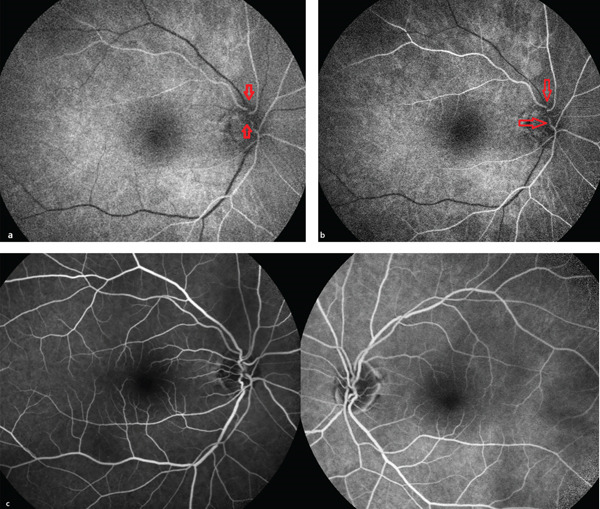
Fundus fluorescein angiography (FFA) of the right and left eyes. (a, b) The right eye FFA reveals two separate arterial circulations in the arterial phase. (c) Venous phase images of the right and left eyes on the FFA.

**Figure 5:**
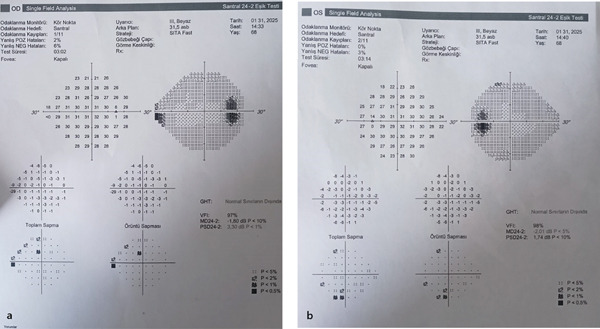
(a, b) Humphrey visual field shows enlarged blind spot in both eyes.

**Figure 6:**
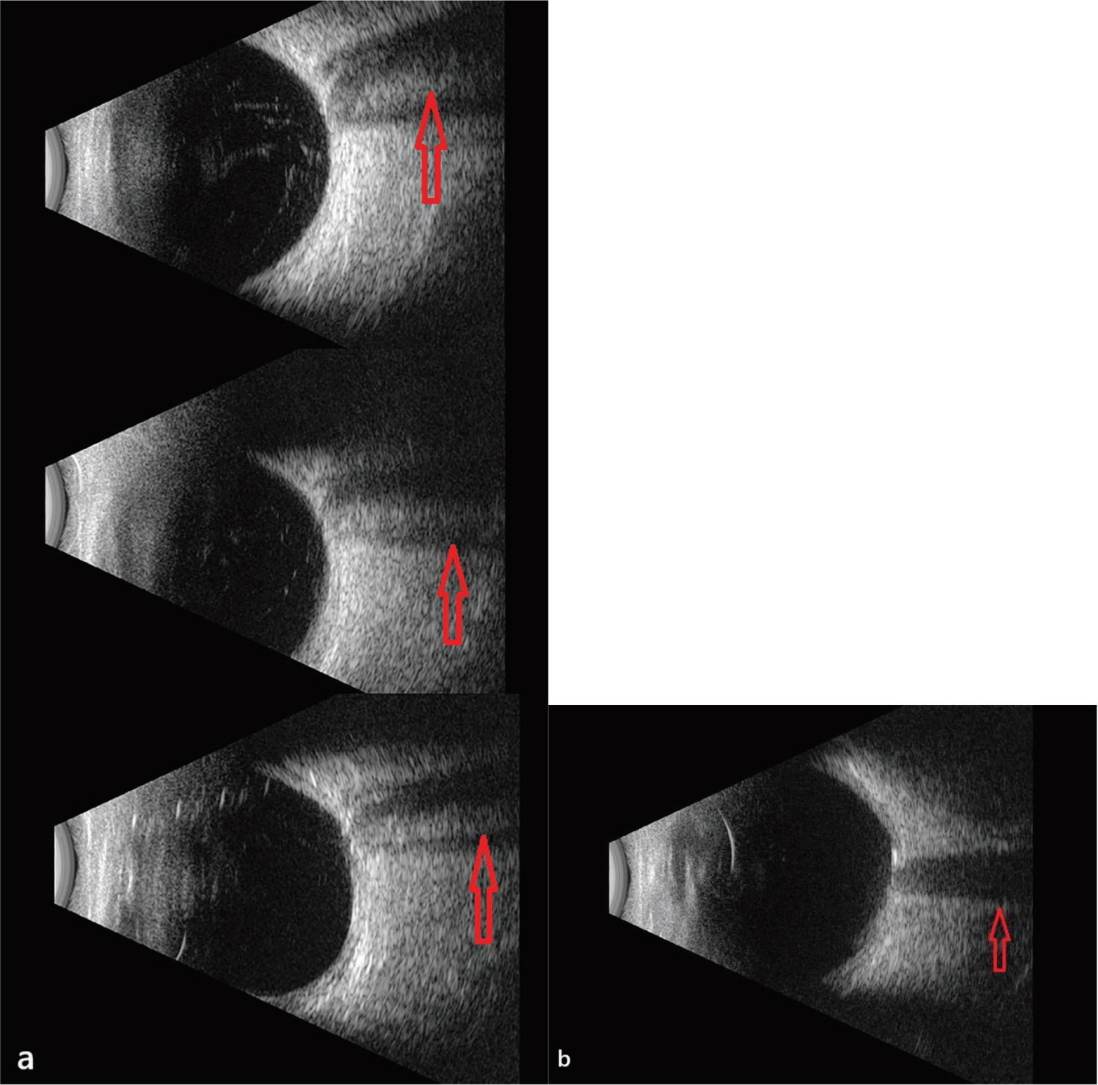
Ocular ultrasonography (USG) of the right and left optic discs. (a) Double optic disc appearance is observed in the right ocular USG, with the separating septum indicated by arrows. (b) Ocular USG of the left eye shows no visible septum.

**Figure 7:**
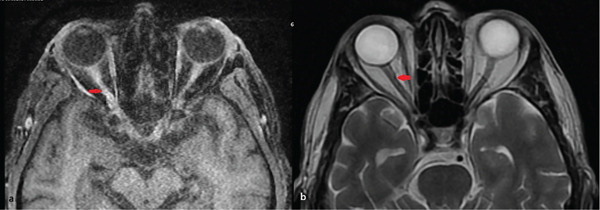
MRI of the right optic nerve. (a–b) MRI of the right optic nerve reveals duplicated optic nerves, with a fusion point marked with an arrow.

A 68‐year‐old male presented to the ophthalmology clinic with complaints of episodic photopsia. The patient′s general health status was unremarkable. His best‐corrected visual acuity (BCVA) was 20/25 in both eyes. Intraocular pressure (IOP) was elevated at 33 mmHg in the right eye and measured 20 mmHg in the left eye. A nuclear cataract, graded as I, was observed. The left pupil was middilated and nonreactive to both direct and consensual light stimulation. Fundoscopic examination of the right eye revealed two distinct sets of retinal blood vessels emerging from the same optic cup (Figure 1). In the fundus photograph of the right eye, a central notch is visible, resembling the fusion of two optic cups (Figure 1). Infrared imaging of the left eye shows a normal optic disc (Figure 2b). Optical coherence tomography analysis of the right optic nerve head reveals two central retinal vessels reflection in comparison to the left eye (Figures 3a, 3b, and 3c). Figure 4a, 4b demonstrates dual arterial filling observed during the arterial phase of fundus fluorescein angiography (FFA) in the right eye. An enlarged blind spot is apparent in the right and left eye in the Humphrey visual field 30‐2 test (Figure 5a, 5b). Ocular ultrasonography (USG) shows the band dividing the optic nerve in the right eye (Figure 6a) and a normal optic nerve in the left eye (Figure 6b). Furthermore, orbital magnetic resonance imaging (MRI) sections revealed the presence of a duplicated optic nerve (Figure 7a, 7b). The patient′s photopsia was considered to be caused by incomplete posterior vitreous detachment. Neuroradiological evaluations revealed no abnormalities that could account for the photopsia. The patient′s photopsia symptoms showed a gradual improvement over time.

## 3. Discussion

True duplication of the optic disc is commonly reported in lower vertebrates but remains an exceptionally rare occurrence in humans [4]. During embryogenesis, the connection between the optic cup and the brain begins in the fourth week, leading to the development of the central retinal artery and optic nerve fibers by the seventh week [5]. Considering this timeline, true optic disc duplication is presumed to originate in early embryogenesis. The division of the optic disc into two separate strands is an expected feature of true duplication, as multidivision of the optic nerve has been documented in certain fish species [2].

Systemic abnormalities such as alopecia and hypogenitalism, along with ophthalmologic anomalies including congenital ptosis and pupillary ectopy, have been reported in cases of double optic disc [2]. The most common cause of pseudodouble optic disc is chorioretinal coloboma, which can be distinguished by the presence of retinal blood vessels traversing the colobomatous area. While the central retinal artery and vein do not typically arise from colobomas, additional retinal vessels may originate from these lesions [6].

For differential diagnosis, ocular USG and MRI can provide valuable insight by demonstrating two distinct optic nerve strands in cases of true optic disc duplication [3].

In conclusion, while a true double optic disc remains exceedingly rare, the presence of two retinal vascular systems arising from the same optic disc represents the rarest morphological form, as seen in the present case. The identification of two distinct arterial filling systems on FFA, together with a separating band in the optic nerve, demonstrated on ocular USG and MRI, serves as strong confirmatory findings for the diagnosis in this case.

## Funding

No funding was received for this manuscript.

## Conflicts of Interest

The authors declare no conflicts of interest.

## Data Availability

The authors confirm that the data are available upon request from the corresponding author. The data are not publicly available due to privacy restrictions.
